# Design, Synthesis, and Biological Evaluation of Phenol Bioisosteric Analogues of 3-Hydroxymorphinan

**DOI:** 10.1038/s41598-019-38911-1

**Published:** 2019-02-19

**Authors:** Ziqiang Li, Xiuqi Bao, Xiaoguang Bai, Guoning Zhang, Juxian Wang, Mei Zhu, Yue Wang, Junmei Shang, Chanjuan Sheng, Dan Zhang, Yucheng Wang

**Affiliations:** 1 School of Pharmacy, Jinzhou Medical University, Jinzhou, Liaoning 121001 China; 2 Institute of Medicinal Biotechnology, Chinese Academy of Medical Science & Peking Union Medical College, Beijing, 100050 China; 3 State Key Laboratory of Bioactive Substrate and Function of Natural Medicine, Institute of Materia Medica, Chinese Academy of Medical Science & Peking Union Medical College, Beijing, 100050 China

## Abstract

The neuroprotective agent 3-hydroxymorphinan (3-HM) is a well-documented and highly safe therapeutic intervention for the inflammatory-related effects of Parkinson’s disease (PD). However, the bioavailability of 3-HM is very low due to the rapid first-pass metabolism of the phenolic moiety. In the present study, we sought to improve the metabolic stability and overall pharmacokinetic profile of 3-HM. Based on an iterative design process that a suitably arranged heterocycle with an NH group would serve as the metabolically stable isostere of the phenolic group, we designed and synthesized two analogues of 3-HM. Benzimidazolone compound **8** (imidazolone-morphinan) was comparable in activity to 3-HM against lipopolysaccharide (LPS)-induced inflammatory responses in microglial BV2 cells and *in vivo* animal experiments (MPTP-induced PD mouse model). Moreover, the *in vitro* study showed that imidazolone-morphinan was non-toxic to microglia, indicating its high safety. Considering the favourable and unique preclinical profiles, compound **8** was nominated as a candidate for further clinical development.

## Introduction

Parkinson’s disease (PD) is a common neurodegenerative disease characterized by deterioration of motor control and is often associated with mood, sleep, attention and cognitive disturbances^1^. It is estimated that approximately 1% of people over the age of 55 suffer from PD^2^. Currently, the therapeutic methods of PD are limited to only symptomatic and supportive treatment but radically fail to stop the progression of the underlying disease. Although levodopa^3,4^ and other drugs such as dopamine agonists^5,6^ can relieve or control the symptoms of the disease, they are often associated with significant and intolerable side effects. Moreover, these drugs cannot prevent the progressive death of dopaminergic neurons^7,8^. Thus, the development of drugs that can prevent dopaminergic neuronal death and slow down disease progression has become the primary goal of PD therapy.

Neuroinflammation is characterized by activated microglia, which play a critical role in forming a self-propelling cycle that leads to sustained chronic neuroinflammation and drives progressive neurodegeneration in PD^9^. Inflammatory mediators such as TNF-α, PGE2, NO, and free radicals as well as other potential products of activated glial cells can also play a role in the degeneration of nigral dopaminergic neurons. Given the central role of neuroinflammation in the pathogenesis of PD^10,11^, treatment for PD has focused on discovering active compounds that can suppress excessive glial activation, which could potentially halt or slow the disease progression.

**Figure 1:**
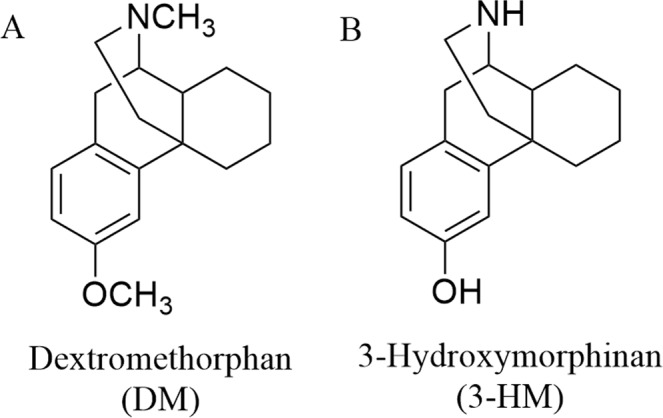
Structures of dextromethorphan and 3-hydroxymorphinan.

Dextromethorphan (DM) (Fig. 1A), an active ingredient in a variety of widely used anti-cough remedies, protects dopaminergic neurons against lipopolysaccharide (LPS)-challenged neuron-glia cultures of the midbrain^12^ and neurotoxin 1-methyl-4-phenyl-1,2,3,6-tetrahydropyridine (MPTP)-elicited neurotoxicity *in vivo*^13^ via an anti-inflammatory effect by preventing the over-activation of microglia. Furthermore, 3-hydroxymorphinan (3-HM) (Fig. 1B), a metabolite of DM, exerts a more potent neuroprotective effect than DM against LPS- and MPTP-elicited dopamine neurotoxicity both *in vivo* and *in vitro*, which is attributed to its additional neurotrophic effect provided by astroglia in addition to the anti-inflammatory activity it shares with DM^13,14^. Mechanistic studies have shown that this neurotrophic effect was due to an increase in the production of several neurotrophic factors by astroglia. Additionally, 3-HM decreased the production of both the extracellular superoxide and intracellular reactive oxygen species (iROS), which may be the basis for the anti-inflammatory mechanism of 3-HM^15^. Thus, these two important features are necessary for the role of 3-HM as an effective neuroprotective agent. In view of the well-documented very low toxicity of 3-HM, 3-HM offers a promising new direction for the development of therapeutic interventions for inflammation-related diseases, such as PD.

**Figure 2:**
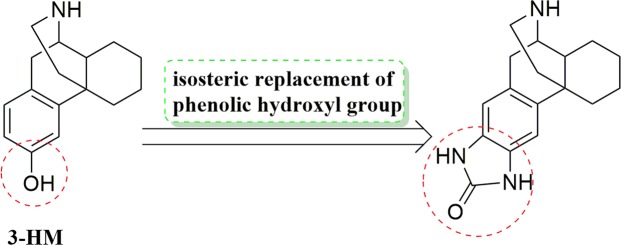
Optimization of 3-hydroxymorphinan.

However, the oral bioavailability of 3-HM is only approximately 18% because of extensive O-glucuronidation of phenol. Therefore, 3-HM is efficacious only if it is administered intraperitoneally or intravenously^16^. As a part of our ongoing programme on the synthesis of a novel neuroprotective drug, we sought to improve the metabolic stability and pharmacokinetic profile of 3-HM. We reasoned that heterocyclic replacements of catechol or phenolic rings would retard the metabolic inactivation due to glucuronidation and hence increase the duration of action. Several examples of the isosteric replacement of one or more phenolic hydroxyl groups with heterocyclic ring systems have been reported^17–22^. We envisioned that a suitably arranged heterocycle with an NH group would serve as a metabolically stable isostere of the phenolic group and improve the pharmacokinetic properties of 3-HM^23^ (Fig. 2). In the present study, we describe the design, synthesis and biological activity of heterocyclic analogues of 3-HM based on an iterative design process. The synthesized compound has potential anti-neuroinflammatory effects *in vitro* and dopaminergic neuroprotection *in vivo* with desirable pharmacokinetic properties and extremely low toxicity, which enable it to be a preclinical drug candidate for the treatment of PD.

## Results and Discussion

### Chemistry

**Figure 3:**
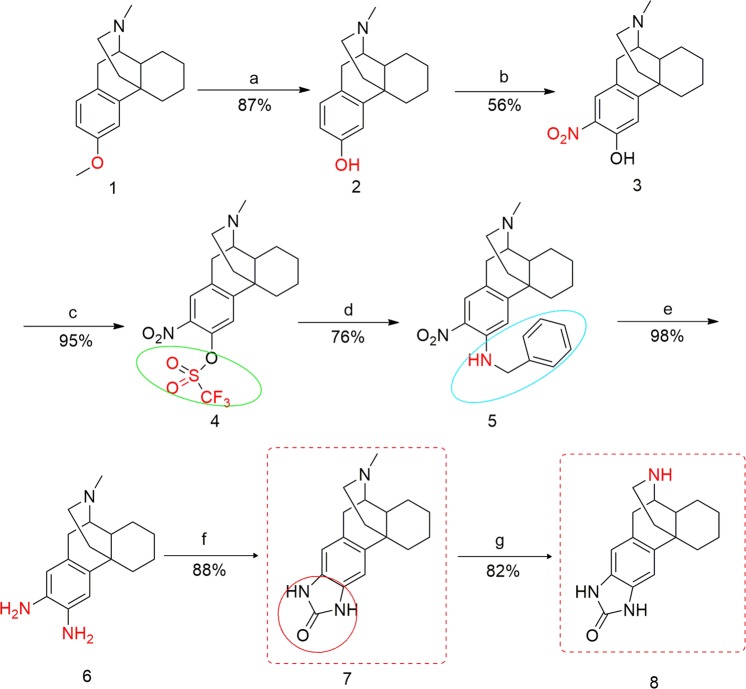
Synthesis of the 3-HM analogue imidazolone-morphinan. Reagents and conditions: (**a**) BBr_3_, CH_2_Cl_2_, −40 °C/rt, overnight; (**b**) 66% HNO_3_, CH_3_COOH, rt, overnight; (**c**) (TfO)_2_O, Et_3_N, CH_2_Cl_2_, 30 min, −15 °C/rt, 8 h; (**d**) BnNH_2_, MeCN, reflux, overnight; (**e**) H_2_, Pd(OH)_2_/C, MeOH, CH_3_COOH, 70 psi, rt, 9 h; (**f**) 1, 1′-carbonyldiimidazole, MeCN, 2 h, rt/reflux, overnight; (**g**) pyridineHCl, MWI, 70 min, 265 °C.

We envisioned a suitably arranged heterocycle with an NH group to serve as a metabolically stable isostere of the phenolic group of 3-HM. Multigram quantities of enantiomerically pure dextromethorphan (DM, 3-methoxy-17-methylmorphinan) **1** were available and served as the starting material as well as the lead compound for our novel targets. The synthesis of the 3-HM analogue imidazolone-morphinan **8** is shown in Fig. 3. Compound **1** was O-demethylated to give the (−)-3-hydroxy-N-methylmorphinan **2**, which, in turn, was nitrated to yield compound **3** as the major isomer. The triflate of alcohol **3** was prepared and heated with benzylamine to give the nitro amine compound **5**. Prolonged catalytic hydrogenation of **5** afforded the diamine intermediate **6** in one pot^23^, which was subsequently treated with 1, 1′-carbonyldiimidazole to form compound **7** in good yield^24^. Compound **7** hydrochloride was N-demethylated with pyridine hydrochloride to produce **8** under microwave irradiation (MWI) conditions.

### Evaluation of neuroprotective activity

#### Effect of 3-HM analogues on nitric oxide (NO) production in an LPS-challenged BV2 cell line

The synthesized 3-HM analogues were screened for their ability to inhibit NO production in LPS-stimulated BV2 cells by measuring nitrite (NO_2_^−^), a stable breakdown product of NO, using the Griess assay. Compound **8** was found to be an effective inhibitor of NO production, with an IC_50_ value of 1.35 μM, which was at the same level as that of 3-HM (IC_50_ value of 1.72 μM). However, very weak inhibition against NO release was found for compound **7**; therefore, compound **8** was selected for further toxicity evaluation. The *in vitro* cell viability assay indicated that compound **8** at 10 μM was non-toxic to cell survival for 48 h of incubation (Table 1).

**Table 1:** Inhibitory effects of compounds **7** and **8** on LPS-induced NO release in BV2 cells and cell viability.

Compound	IC_50_ (μM)	Cell viability (%)
7	11.8	ND
8	1.35	100
3-HM	1.72	100

ND: not detected.

**Figure 4:**
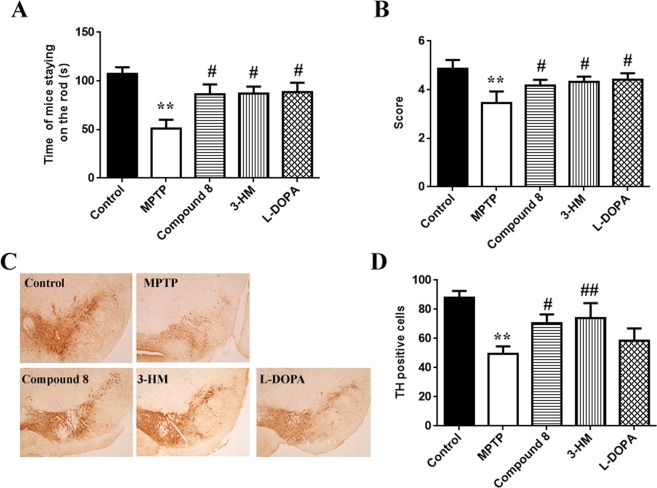
The effects of compound **8** on the sub-acute PD mouse model induced by MPTP. Mice were injected with MPTP hydrochloride (30 mg/kg, i.p.) for 5 consecutive days; compound **8** (50 mg/kg), 3-HM (50 mg/kg) or L-DOPA (20 mg/kg) were administered 30 min before each MPTP injection, and administration continued for the next 7 days. Compound **8** improved motor behaviour of mice, as measured by (**A**) rotarod test and (**B**) pole test (n = 15). (**C**,**D**) TH-positive staining neurons. Representative sections of substantia nigra from 5 mice are shown. Data were the mean ± SEM; **P < 0.01 *versus* control mice; ^#^*P* < 0.05, ^##^*P* < 0.01 *versus* MPTP–injected mice.

Therapeutic effects of the 3-HM analogue imidazolone-morphinan on the MPTP-induced sub-acute PD mouse model. Considering the efficacy of compound **8** in suppressing NO release and with no toxicity to cells, the compound was then subjected to an *in vivo* study in the MPTP-induced PD mouse model. The results showed that compound **8** exhibited greatly improved motor behaviour dysfunction of the mice by increasing the staying time on the rod in the rotarod tests and the performance score in the pole tests, which was equivalent to that of 3-HM or L-DOPA (Fig. 4A,B). Furthermore, compound **8** significantly increased TH-positive neurons in the substantia nigra of PD mice, and this effect was superior to that of L-DOPA (Fig. 4C,D). This *in vivo* study demonstrated the potent neuroprotective effects of compound **8** on dopaminergic neurons, which might be associated with the activity of suppressing neuroinflammation.

## Conclusion

In summary, we applied a rational design approach to improve on the metabolic stability of 3-HM. To this end, imidazolone-morphinan, which contains an N-H hydrogen bond donor, was synthesized. Our findings demonstrated that imidazolone-morphinan exerted neuroprotective effects on dopaminergic neurons in a PD mouse model induced by MPTP, which might be related to the anti-neuroinflammatory effects. The principles of phenolic isostere replacement demonstrated here may benefit future investigations. This study also provided evidence that imidazolone-morphinan might be a potential drug candidate for the treatment of PD. Extensive preclinical evaluation for the pharmacokinetic properties of imidazolone-morphinan is actively underway, and the results will be reported in due course.

## Methods

### Synthetic procedure and analytical data of imidazolone-morphinan and the reaction intermediates (1–8)

#### Methods and Materials

^1^H and ^13^C NMR spectra were measured on a Bruker or Varian 400 or 500 MHz NMR spectrometer. Melting points were measured on a Mettler Toledo capillary melting point apparatus and are uncorrected. Specific rotations were measured on a RUDOLPH AUTOPOL IV-T. ESI high-resolution mass spectra (HRMS) was recorded on an Autospec Ultima-TOF spectrometer. A CEM Discovery Microwave Reactor was used for microwave synthesis. All reactions were monitored by thin layer chromatography (TLC) carried out on Merck silica-gel plates (0.25 mm thick, 60 F254), visualized by using UV (254 nm) or dyes such as ninhydrin or phosphomolybdic acid. Buffer salts (reagent grade or better), solvents (HPLC grade or better), and all the other chemicals were purchased from Fisher Scientific Co. The chemicals were purchased from Aldrich Co. and Sigma and purified before use by standard methods.

### Synthesis of dextrorphan (**2**)

BBr_3_ (191.30 g, 0.76 mol) in dichloromethane (150 mL) was added dropwise at −30 °C to a solution of DM (**1**) (34.54 g, 0.13 mmol) in dichloromethane (180 mL) under a nitrogen atmosphere^25^. Then, the mixture was stirred at room temperature overnight. After completion, the reaction mixture was then poured into a well-stirred mixture of 1000 g of ice and 255 mL of concentrated ammonium hydroxide (28–30% NH_3_), and the pH of the solution was adjusted to pH 8 using NH_4_OH. The aqueous layer was extracted with CH_2_Cl_2_ (300 mL × 3), and the combined organic extracts were dried over Na_2_SO_4_, then filtered and concentrated. The crude product was purified by silica gel (CH_2_Cl_2_:MeOH (1–10%)) to give a light yellow solid **2** (29.11 g, 87% yield) (mp = 216–217 °C); ${[{\rm{\alpha }}]}_{{\rm{D}}}^{20}$ + 36.36^°^ (c = 0.0660, CHCl_3_); ^1^H NMR (500 MHz, CDCl_3_) δ 6.96 (d, *J* = 8.0 Hz, 1 H), 6.72 (d, *J* = 2.5 Hz, 1 H), 6.61 (dd, *J* = 8.5, 2.5 Hz, 1 H), 2.97 (d, *J* = 18.0 Hz, 1 H), 2.88 (s, 1 H), 2.65 (dd, *J* = 18.0, 5.5 Hz, 1 H), 2.51 (dd, *J* = 12.5, 3.5 Hz, 1 H), 2.42 (s, 3 H), 2.31–2.26 (m, 1 H), 2.18 (td, *J* = 12.5, 3.0 Hz, 1 H), 1.90 (d, *J* = 12.5 Hz, 1 H), 1.78 (td, *J* = 12.5, 4.5 Hz, 1 H), 1.63 (d, *J* = 10.5 Hz, 1 H), 1.50 (s, 1 H), 1.41 (d, *J* = 13.0 Hz, 1 H), 1.37–1.26 (m, 4 H), 1.15 (qd, *J* = 12.0, 3.5 Hz, 1 H); ^13^C NMR (126 MHz, CDCl_3_) δ 156.16, 139.87, 128.90, 125.43, 114.11, 112.21, 59.30, 47.52, 42.42, 41.54, 39.62, 36.33, 35.64, 26.18, 25.86, 24.04, 21.85. HRMS (ESI) m/z calcd. for C_17_H_23_ONH^+^: 258.18524 Found: 258.18467.

### Synthesis of 2-Nitro-3-hydroxyl-N-methylmorphinan (**3**)

A solution of 14.90 mL 66% nitric acid (0.22 mol) in 15 mL of acetic acid was slowly added to a solution of dextrorphan **2** (29 g, 0.11 mol) in 15 mL acetic acid under a nitrogen atmosphere^23^. The mixture was stirred at room temperature overnight. After completion, the mixture was made basic with saturated sodium bicarbonate and extracted with methylene chloride. The combined organic extracts were washed with brine and concentrated. The residue was chromatographed eluting with CH_2_Cl_2_:MeOH (1–10%) to give 18.61 g (56% yield) of compound **3** as yellow solid (mp = 127–128 °C); ^1^H NMR (500 MHz, CDCl_3_) δ 7.86 (s, 1 H), 7.06 (s, 1 H), 3.06 (d, *J* = 18.5 Hz, 1 H), 2.91–2.87 (m, 1 H), 2.65 (dd, *J* = 18.0, 5.5 Hz, 1 H), 2.51 (dd, *J* = 11.0, 3.5 Hz, 1 H), 2.42 (s, 3 H), 2.35 (dd, *J* = 8.5, 6.5 Hz, 1 H), 2.06 (td, *J* = 12.5, 3.0 Hz, 1 H), 1.90 (dd, *J* = 13.0, 3.0 Hz, 1 H), 1.84 (td, *J* = 13.0, 4.5 Hz, 1 H), 1.68 (d, *J* = 13.0 Hz, 1 H), 1.58 (d, *J* = 13.0 Hz, 1 H), 1.50–1.45 (m, 1 H), 1.43–1.34 (m, 3 H), 1.27–1.17 (m, 2 H), 1.03 (qd, *J* = 12.5, 3.5 Hz, 1 H); MS (ESI) m/z 272.2 (M + 1)^+^.

### Synthesis of 2-Nitro-N-methyl-3-trifluoromethanesulfonate morphinan ester (**4**)

A solution of 17.26 g (91.77 mmol) of trifluoromethanesulfonic anhydride in 50 mL of methylene chloride at −15 °C was added to a solution of compound **3** (18.50 g, 61.18 mmol) and 25.51 mL (183.54 mmol) of triethylamine in 300 mL of methylene chloride under an argon atmosphere^23^. The mixture was warmed to room temperature and stirred overnight. Upon completion of the reaction, the solution was diluted with 200 mL of methylene chloride and washed with 300 mL of saturated sodium bicarbonate. The aqueous layer was extracted with three 150 mL portions of methylene chloride. The combined organic extracts were washed with brine and concentrated. The residue was chromatographed eluting with 90% to 100% ethyl acetate in petroleum ether to give 25.25 g (95% yield) of compound **4** as yellow solid (mp = 105–106 °C); ${[{\rm{\alpha }}]}_{{\rm{D}}}^{20}$ + 39.86^°^ (c = 0.1380, CHCl_3_); ^1^H NMR (500 MHz, CDCl_3_) δ 7.97 (s, 1 H), 7.28 (s, 1 H), 3.16 (d, *J* = 19.0 Hz, 1 H), 2.91 (dd, *J* = 5.5, 3.0 Hz, 1 H), 2.71 (dd, *J* = 19.0, 5.5 Hz, 1 H), 2.55–2.49 (m, 1 H), 2.41 (s, 3 H), 2.31 (d, *J* = 14.5 Hz, 1 H), 2.00–1.91 (m, 2 H), 1.87 (td, *J* = 12.5, 4.5 Hz, 1 H), 1.70 (d, *J* = 13.0 Hz, 1 H), 1.62 (d, *J* = 13.5 Hz, 1 H), 1.49 (td, *J* = 14.0, 3.5 Hz, 2 H), 1.40 (dt, *J* = 13.0, 3.5 Hz, 1 H), 1.36–1.31 (m, 1 H), 1.19–1.08 (m, 1 H), 0.99 (qd, *J* = 13.0, 4.0 Hz, 1 H); HRMS (ESI) m/z calcd. for C_18_H_21_O_5_N_2_F_3_SH^+^: 435.11960 Found: 435.11920.

### Synthesis of 2-nitro-3-benzylamino-N-methylmorphinan (**5**)

A solution of benzylamine (18.50 g, 172.64 mL) in 60 mL acetonitrile was added to a solution of compound **4** (25.00 g, 57.55 mmol) in 270 mL acetonitrile under an argon atmosphere at reflux^23^. The mixture was stirred at reflux overnight. After completion, the mixture was diluted with 300 mL of methylene chloride and washed with 300 mL of saturated sodium bicarbonate. The aqueous layer was extracted with two 200 mL portions of methylene chloride. The combined organic extracts were washed with brine, dried over Na_2_SO_4_, filtered, and concentrated to give a crude solid product which was purified by gradient silica gel flash chromatography (ethyl acetate:petroleum ether 1:2 → 4:5) to give compound **5** (17.12 g, 76% yield) as a brownish solid (mp = 57–58 °C); ${[{\rm{\alpha }}]}_{{\rm{D}}}^{20}$ + 43.06^°^ (c = 0.0720, CHCl_3_); ^1^H NMR (500 MHz, CDCl_3_) δ 8.31 (t, *J* = 5.5 Hz, 1 H), 7.94 (s, 1 H), 7.38–7.32 (m, 4 H), 6.63 (s, 1 H), 4.53 (ddd, *J* = 38.5, 18.0, 6.0 Hz, 2 H), 3.48 (s, 1 H), 2.99 (d, *J* = 18.0 Hz, 1 H), 2.81 (d, *J* = 3.0 Hz, 1 H), 2.56 (dd, *J* = 18.0, 5.5 Hz, 1 H), 2.44 (dd, *J* = 12.0, 3.5 Hz, 1 H), 2.39 (s, 3 H), 2.05 (ddd, *J* = 23.5, 14.0, 3.0 Hz, 2 H), 1.75 (ddd, *J* = 23.0, 15.0, 5.0 Hz, 2 H), 1.57 (dd, *J* = 10.0, 1.5 Hz, 1 H), 1.43–1.38 (m, 1 H), 1.31–1.20 (m, 4 H), 0.99 (qd, *J* = 12.5, 4.0 Hz, 1 H), 0.89–0.79 (m, 1 H); HRMS (ESI) m/z calcd. for C_24_H_29_O_2_N_3_H^+^: 392.23325 Found: 392.23251.

### Synthesis of N-methylmorphinan-2,3-diamine (**6**)

A mixture of **5** (17.01 g, 43.45 mmol), acetic acid (23.16 mL, 130.35 mmol), 20% Pd/C (8.54 g, 60.83 mmol) and CH_3_OH (300 mL) was subjected to 60 psi H_2_ in a hydrogenation bottle at 25 °C for 7 h. As indicated by TLC, all starting material was consumed, and the mixture was filtered and concentrated to give a viscous product. The product was made basic with saturated NaHCO_3_ and concentrated to give a brownish solid product. The brownish product was dissolved in chloroform:isopropanol 3:1, filtered and concentrated to give compound **6** (11.56 g, 98% yield) as a brownish solid (mp = 100–101 °C); ${[{\rm{\alpha }}]}_{{\rm{D}}}^{20}$ + 54.24° (c = 0.2280, CHCl_3_); ^1^H NMR (500 MHz, CDCl_3_) δ 6.58 (s, 1 H), 6.46 (s, 1 H), 3.32 (s, 4 H), 2.87 (d, *J* = 18.0 Hz, 1 H), 2.78 (dd, *J* = 5.5, 3.5 Hz, 1 H), 2.52 (dd, *J* = 18.5, 6.0 Hz, 1 H), 2.43 (dd, *J* = 11.5, 5.0 Hz, 1 H), 2.39 (s, 3 H), 2.29–2.22 (m, 1 H), 2.14 (td, *J* = 12.0, 3.0 Hz, 1 H), 1.77 (dt, *J* = 12.0, 2.5 Hz, 1 H), 1.69 (td, *J* = 12.5, 5.0 Hz, 1 H), 1.63 (dd, *J* = 7.5, 3.5 Hz, 1 H), 1.51–1.46 (m, 1 H), 1.40–1.29 (m, 4 H), 1.28–1.25 (m, 1 H), 1.22–1.12 (m, 1 H); ^13^C NMR (126 MHz, CDCl_3_) δ 133.85, 133.19, 130.60, 127.19, 115.72, 113.55, 58.73, 47.30, 43.38, 41.59, 40.58, 36.08, 35.88, 26.38, 26.26, 24.05, 22.09. HRMS (ESI) m/z calcd. for C_17_H_25_N_3_H^+^: 272.21212 Found: 272.21124.

### Synthesis of imidazolone N-methylmorphinan (**7**)

Diamine **6** (11.00 g, 40.53 mmol) was added to 1, 1′-carbonyldiimidazole (29.57 g, 182.38 mmol) in acetonitrile (180 mL)^24^. The mixture was stirred at room temperature for 2 h and then refluxed overnight under an argon atmosphere. After completion of the reaction, the solvent was evaporated, and the residue was purified by column chromatography (methanol:ethyl acetate 1:100 → 1:10) to give compound **7** (10.61 g, 88% yield) as a white solid (mp = 312–313 °C); ${[{\rm{\alpha }}]}_{{\rm{D}}}^{20}$ + 30.70^°^ (c = 0.1140, MeOH); ^1^H NMR (500 MHz, CDCl_3_) δ 9.79 (s, 2 H), 6.97 (s, 1 H), 6.84 (s, 1 H), 3.05 (d, *J* = 18.0 Hz, 1 H), 2.85 (s, 1 H), 2.72 (dd, *J* = 18.0, 5.0 Hz, 1 H), 2.48 (d, *J* = 10.5 Hz, 1 H), 2.42 (s, 3 H), 2.32 (d, *J* = 13.0 Hz, 1 H), 2.09 (t, *J* = 11.0 Hz, 1 H), 1.87 (d, *J* = 12.5 Hz, 1 H), 1.78 (dd, *J* = 12.5, 8.5 Hz, 1 H), 1.63 (d, *J* = 11.0 Hz, 1 H), 1.49 (d, *J* = 12.0 Hz, 1 H), 1.42 (d, *J* = 12.0 Hz, 1 H), 1.30 (dt, *J* = 42.5, 12.5 Hz, 4 H), 1.13 (dt, *J* = 22.0, 12.0 Hz, 1 H); ^13^C NMR (126 MHz, CDCl_3_) δ 157.26, 133.85, 130.99, 128.27, 127.30, 108.37, 106.26, 57.96, 47.19, 45.19, 42.65, 42.22, 37.06, 36.93, 26.70, 26.54, 24.53, 22.14. HRMS (ESI) m/z calcd. for C_18_H_23_ON_3_H^+^: 298.19139. Found: 298.19114.

### Synthesis of imidazolone-morphinan (**8**)

A mixture of **7** (5.00 g, 16.82 mmol) and pyridine hydrochloric acid (13.61 g, 117.77 mmol) was heated at 265 °C in the microwave for 70 min. After completion, the reaction mixture was poured into concentrated ammonium hydroxide (28–30% NH_3_), and the pH of the solution was adjusted to pH 10 using NH_4_OH. The aqueous layer was extracted with chloroform:isopropanol 3:1 (150 mL × 3), and the combined organic extracts were dried over Na_2_SO_4_, then filtered and concentrated. The crude product was purified over RP C-18 silica gel (MeOH:H_2_O 55:45) to give light yellow solid **8** (3.91 g, 82% yield) (mp = 302–303 °C); ${[{\rm{\alpha }}]}_{{\rm{D}}}^{20}$ + 16.67° (c = 0.1680, MeOH); ^1^H NMR (500 MHz, DMSO-*d*_6_) δ 10.36 (s, 2 H), 6.73 (s, 1 H), 6.63 (s, 1 H), 3.03 (dd, *J* = 17.5, 5.0 Hz, 1 H), 2.89 (s, 1 H), 2.69 (d, *J* = 17.5 Hz, 1 H), 2.40 (t, *J* = 12.0 Hz, 1 H), 2.23 (d, *J* = 13.0 Hz, 1 H), 1.65 (d, *J* = 11.0 Hz, 1 H), 1.56 (s, 1 H), 1.45 (d, *J* = 11.5 Hz, 2 H), 1.36–1.22 (m, 4 H), 1.16 (d, *J* = 11.5 Hz, 2 H), 1.07–0.80 (m, 2 H); ^13^C NMR (126 MHz, DMSO) δ 155.59, 130.35, 129.19, 128.34, 127.74, 107.44, 104.84, 49.80, 42.19, 37.15, 36.52, 36.11, 30.04, 25.85, 25.49, 23.71, 21.57. HRMS (ESI) m/z calcd. for C_17_H_21_ON_3_H^+^: 284.17574. Found: 284.17556.

### Biological assays

*BV-2 cell culture*, *treatment and NO production assay*. Under an atmosphere of 5% CO_2_, BV-2 cells were cultured at 37 °C in Dulbecco’s modified Eagle medium (DMEM) supplemented with 10% foetal bovine serum (FBS), 100 U/mL penicillin, and 100 μg/mL streptomycin. For the compound screening experiments, the cells were plated in 96-well plates at a density of 5 × 10^3^ cells/well and 24 h later were treated with compounds **7**, **8** or 3-HM at six different concentrations (100, 50, 10, 5, 1 and 0.1 µM). One hour later the cells were treated with 300 ng/mL of LPS (Sigma, USA). After incubation for another 24 h, the culture media was collected for the detection of the NO concentration. The NO accumulation in the medium was determined by measuring the production of nitrite (NO_2_^−^) using the Griess Reagent Kit (Sigma, USA).

#### Cell viability assay

Cell viability was measured by the MTT reduction assay. BV2 cells (5 × 10^3^ cells/well in 96-well plates) were incubated with compounds **8** or 3-HM at the concentration of 10 µM for 48 h. Then, 100 μL of MTT at a concentration of 0.5 mg/mL was added to each well and incubated at 37 °C for 4 h. The supernatant was discarded and 200 μL of DMSO was added to each well. The optical density of each well was determined at 490 nm using a Microplate Reader.

#### Development of the PD mouse model and drug administration

Male C57/BL mice (Animal Center of the Chinese Academy of Medical Sciences) weighing 22–25 g were used to establish the MPTP-induced PD models. The mice were maintained on a 12 h light/dark cycle at 24 °C in a room with a relative humidity of 60%. The animals were provided with food and water *ad libitum* and allowed to adapt to the conditions for 1 week before the experimentation. The mice were injected with MPTP (30 mg/kg of MPTP hydrochloride, intraperitoneally, i.p.) consecutively for 5 days. Compound **8** (50 mg/kg), L-DOPA (20 mg/kg, suspended in 0.5% sodium carboxymethylcellulose (CMC-Na), oral administration) or 3-HM (50 mg/kg, dissolved in normal saline, i.p.) were administered 30 min before each MPTP injection, and compound **8**, L-DOPA or 3-HM were continually administered to the mice for the next 7 days after the last injection of MPTP. Control mice were treated with 0.5% CMC-Na. All experiments were performed in accordance with the guidelines of the Beijing Municipal Ethics Committee for the Care and Use of Laboratory Animals.

#### Rotarod test

The rotarod test, which requires animals to balance and walk on a rotating cylinder, is used to measure coordinated motor skills. The mice were positioned on the rotarod, which was then set to revolve at 14 rpm for up to 120 s. The rotarod automatically recorded the time when the animals first fell off the rod, which was designated as the latency. The mice were tested in triplicate, and the latency was recorded each time. The animals were allowed to rest for 1 h between each trial.

#### Pole test

The pole used in this study was constructed of wood with height of 50 cm and diameter of 3 cm and wrapped in gauze to prevent slipping, while the base was positioned in the home cage. A wooden ball was glued to the top of the pole to prevent animals from sitting on the top and to help position the animals on the pole. The performance of the mice while they descended the pole was then scored on a scale of 1 to 5, with 1 being the lowest score. If the mouse did not descend within 60 s, it was guided down. The mice were pre-trained before the experiment, and they each performed two successive trials, with a 1 h interval between the trials.

#### Histochemical analysis

The TH immunohistochemical analysis was performed as previously described^26^. Briefly, the brains of the mice were fixed and cut into 40 μm sections using a freezing microtome and the coronal sections through the substantia nigra were processed. The sections were incubated with primary antibodies against TH (Abcam, USA) and the labelled proteins were visualized using 0.04% hydrogen peroxidase and 0.05% 3,3′-diaminobenzidine. The sections were observed using a light microscope (NIKON E600, Japan), and the number of positively stained cells in each group was recorded. All quantifications were performed blindly.

#### Statistical analysis

The data are expressed as the mean ± SEM and were analysed using a one-way analysis of variance (ANOVA) followed by Dunnett’s post hoc test. The results were considered statistically significant at *P* < 0.05.

## Supplementary information

MS and NMR spectra
