# Supplementary material for: Design, Synthesis, and Biological Evaluation of Phenol Bioisosteric Analogues of 3-Hydroxymorphinan
Source: Sci Rep. 2019 Feb 19;9:2247. doi: 10.1038/s41598-019-38911-1 (PMC6381151; doi:10.1038/s41598-019-38911-1)
Supplement: Supplementary file 1 — MS and NMR spectra [file 41598_2019_38911_MOESM1_ESM.pdf]

Design, Synthesis, and Biological Evaluation of Phenol Bioisosteric Analogues of 3-Hydroxymorphinan

Ziqiang Li[1,+], Xiuqi Bao[3,+], Xiaoguang Bai[2], Guoning Zhang[2], Juxian Wang[2], Mei Zhu[2], Yue Wang[3], Junmei Shang[3], Chanjuan Sheng[3], Dan Zhang[3,*] & Yucheng Wang[2,*]

[1]School of Pharmacy, Jinzhou Medical University, Jinzhou, Liaoning 121001, China.

[2]Institute of Medicinal Biotechnology, Chinese Academy of Medical Science & Peking Union Medical College, Beijing 100050, China.

[3]State Key Laboratory of Bioactive Substrate and Function of Natural Medicine, Institute of Materia Medica, Chinese Academy of Medical Science & Peking Union Medical College, Beijing 100050, China.

[+]These authors contributed equally to this work. Correspondence and requests for materials should be addressed to Y.W. (email: wangyucheng@imb.pumc.edu.cn) or D.Z. (email: danzhang@imm.ac.cn)

MS and NMR spectra of synthesized imidazolone-morphinan and intermediates

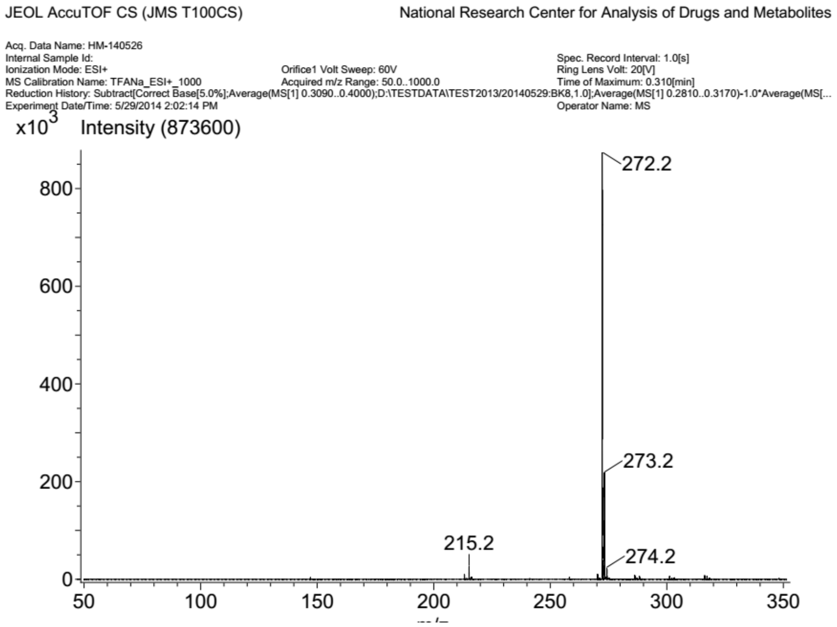

*MS spectra of compound dextromethorphan (**1**)*

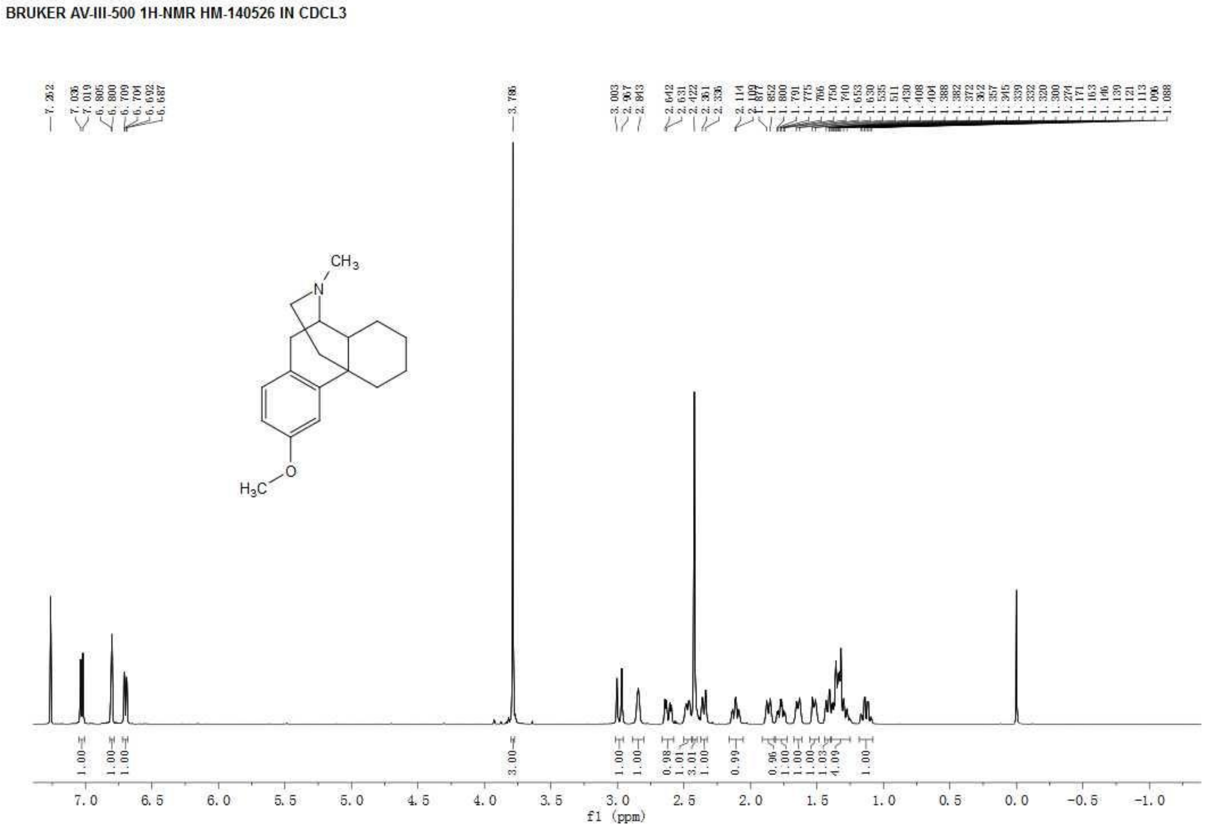

*^{1}H NMR spectra of compound dextromethorphan (**1**)*

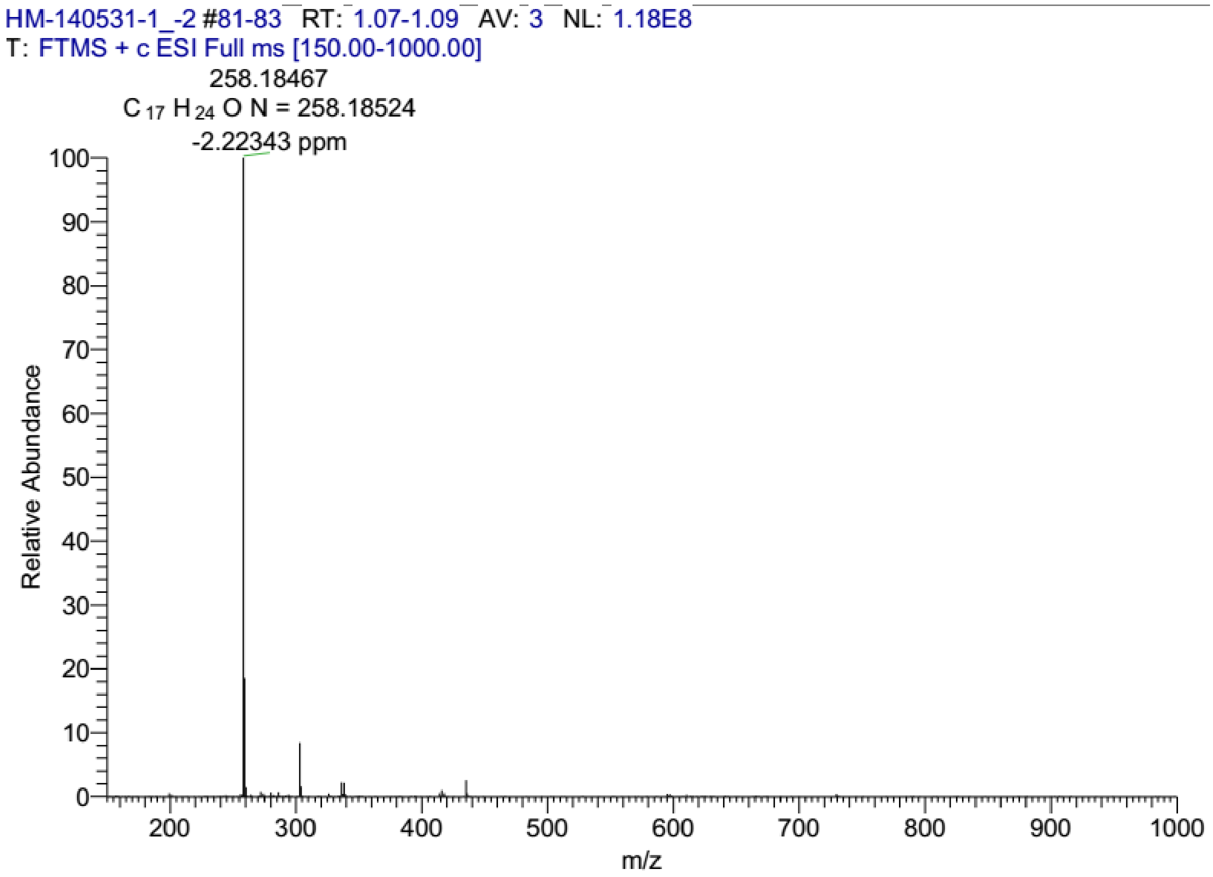

*HRMS spectra of compound dextrorphan (**2**)*

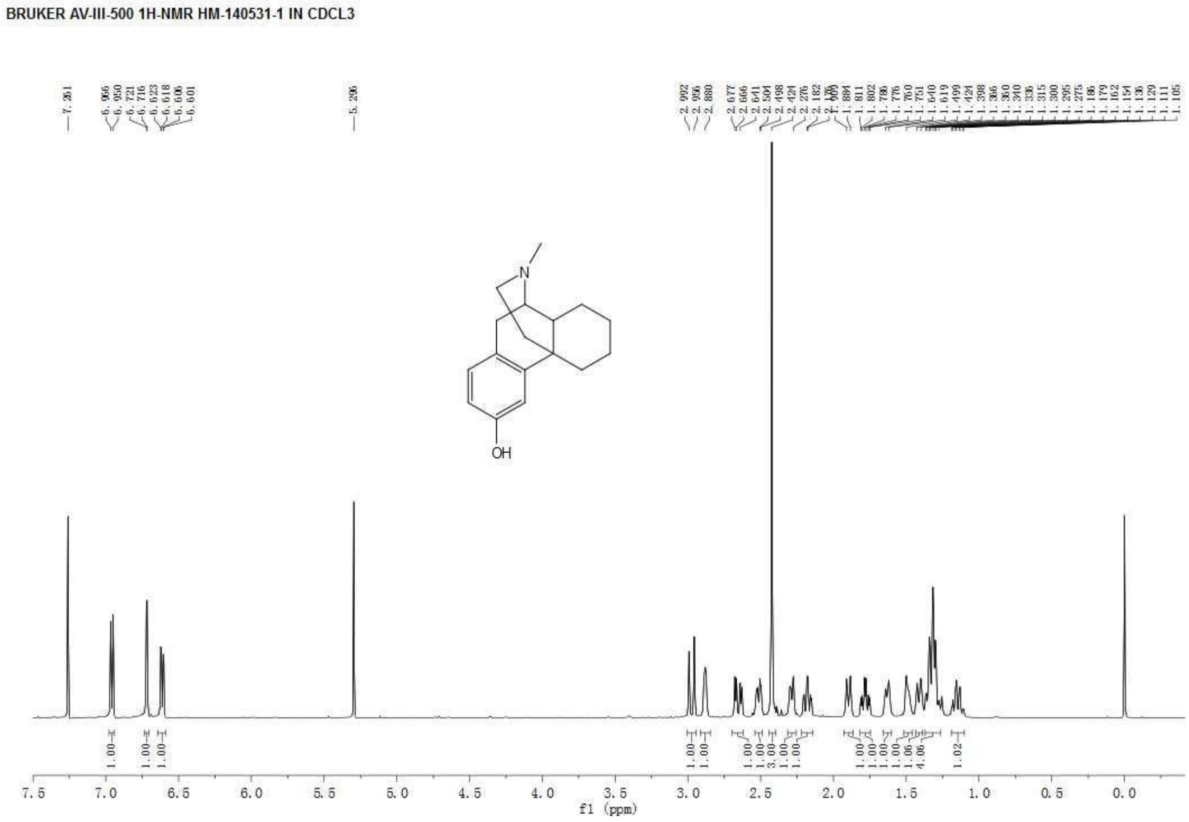

*^{1}H NMR spectra of compound dextrorphan (**2**)*

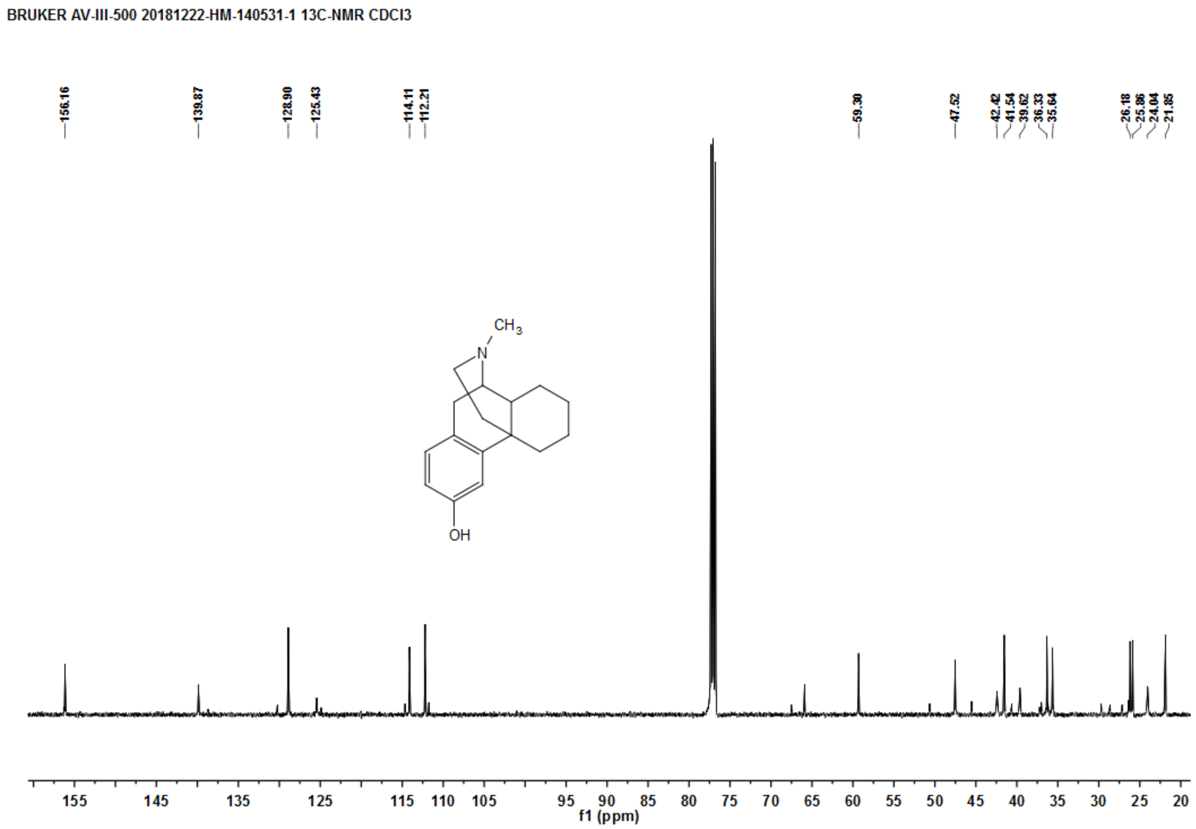

^{13}*C NMR spectra of compound dextrorphan (**2**)*

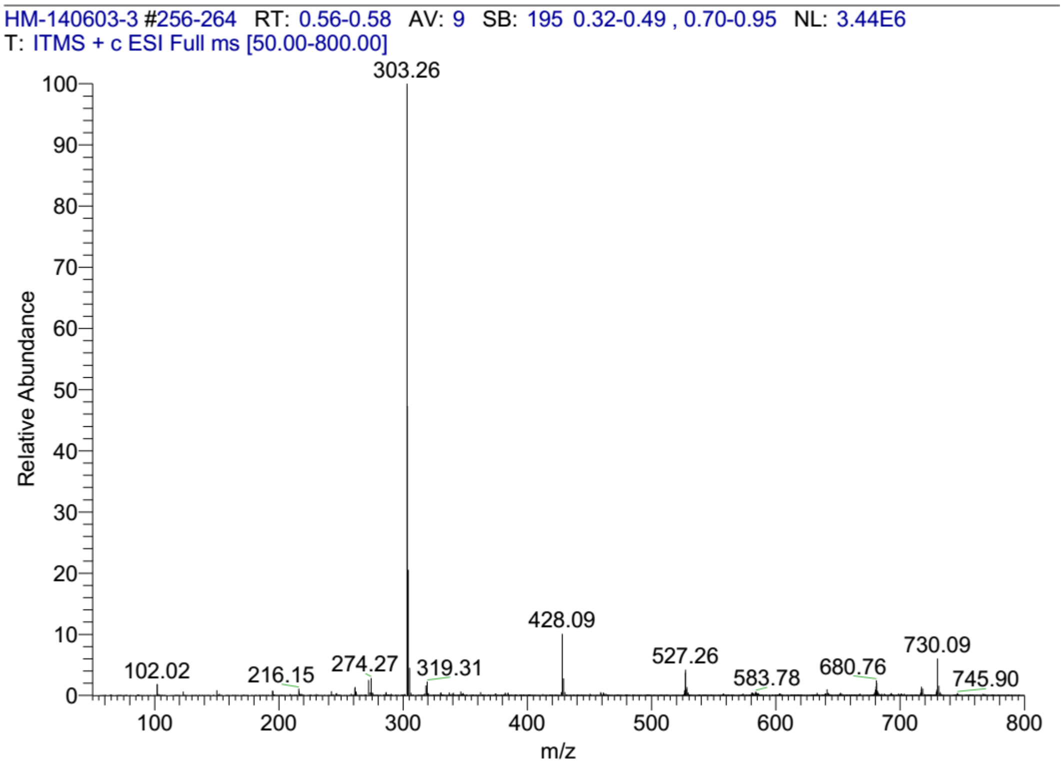

*MS spectra of compound 2-nitro-3-hydroxyl-N-methylmorphinan (**3**)*

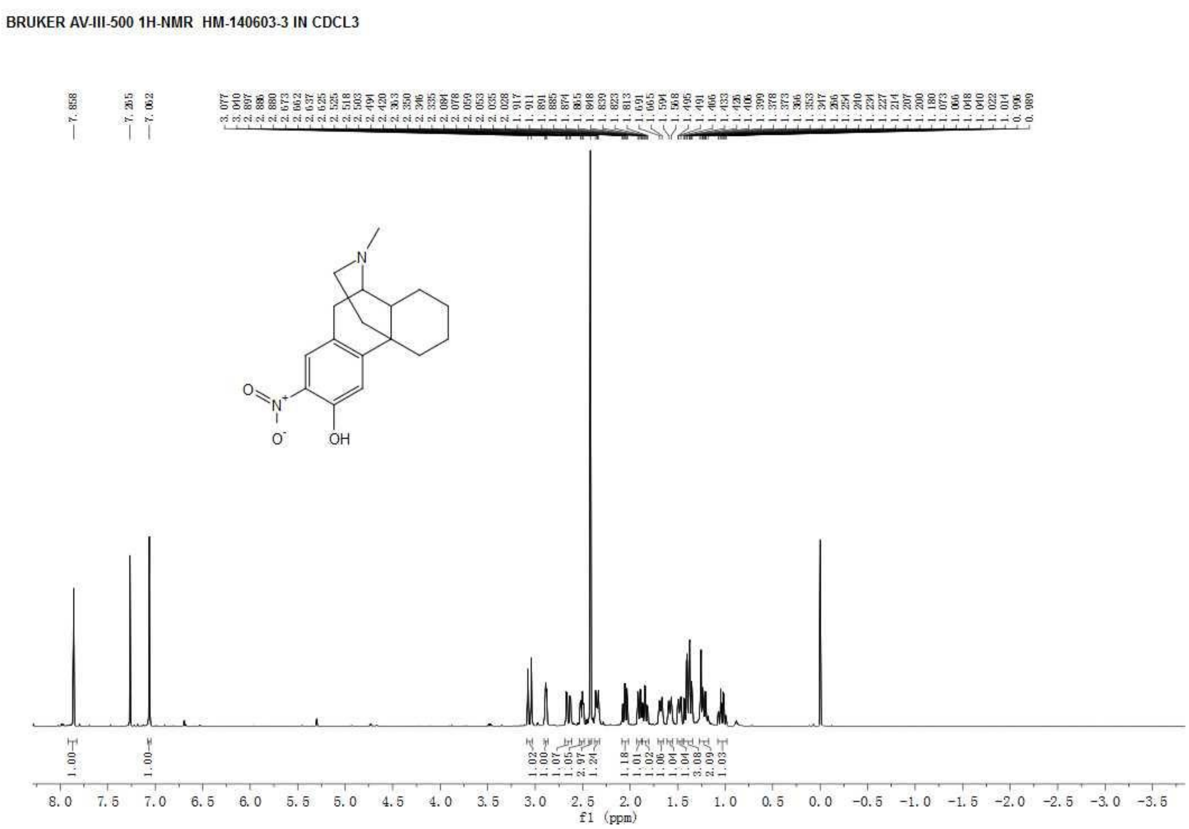

*^{1}H NMR spectra of compound 2-nitro-3-hydroxyl-N-methylmorphinan (**3**)*

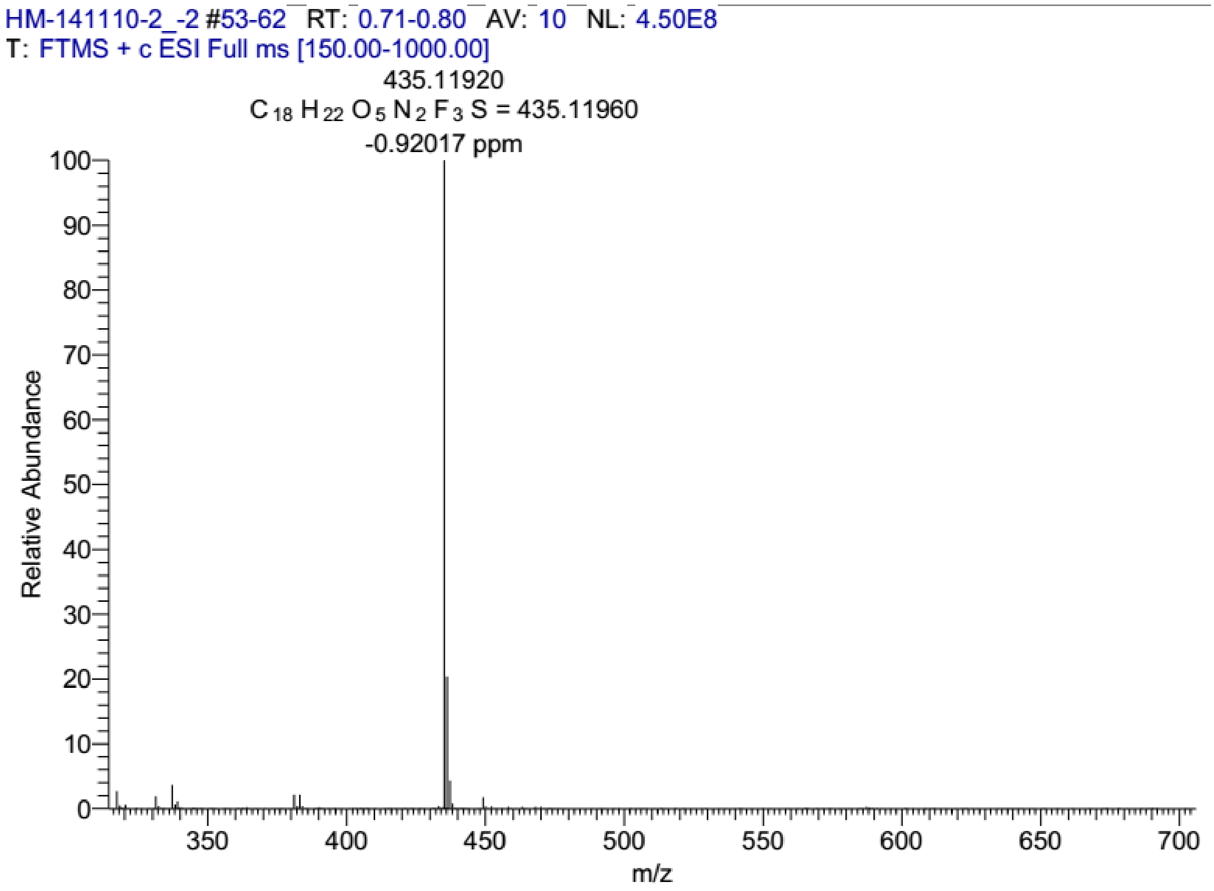

*HRMS spectra of compound 2-Nitro-N-methyl-3-trifluoromethanesulfonatemorphinan ester (**4**)*

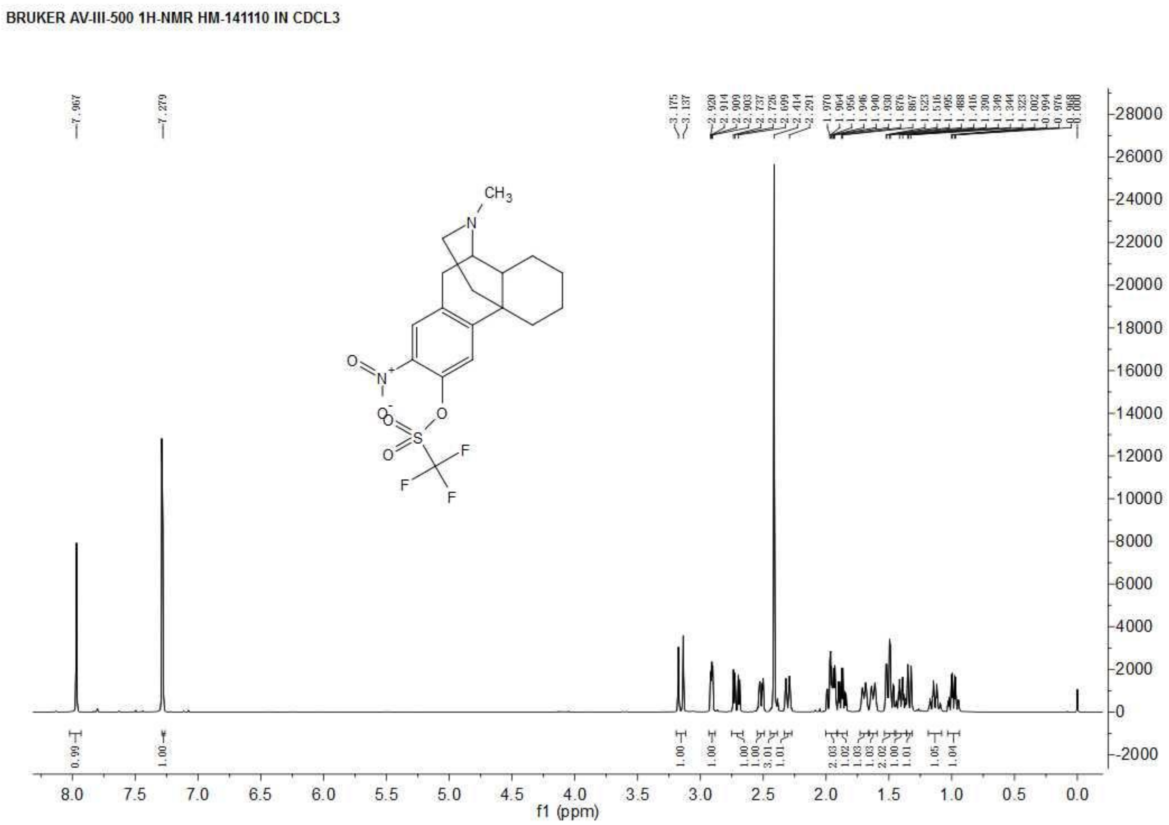

^{1}H NMR spectra of compound 2-Nitro-N-methyl-3-trifluoromethanesulfonatemorphinan ester (**4**)

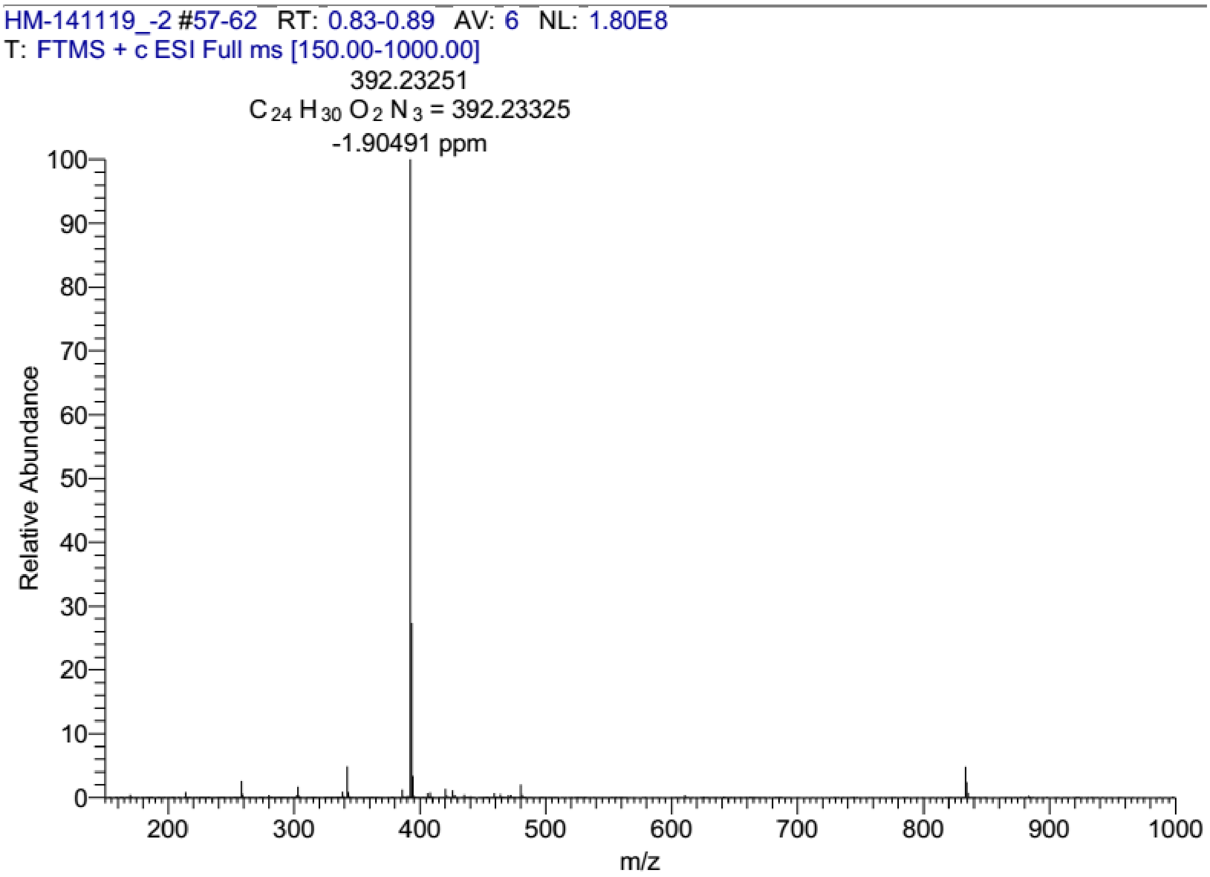

*HRMS spectra of compound 2-nitro-3-benzylamino-N-methylmorphinan (**5**)*

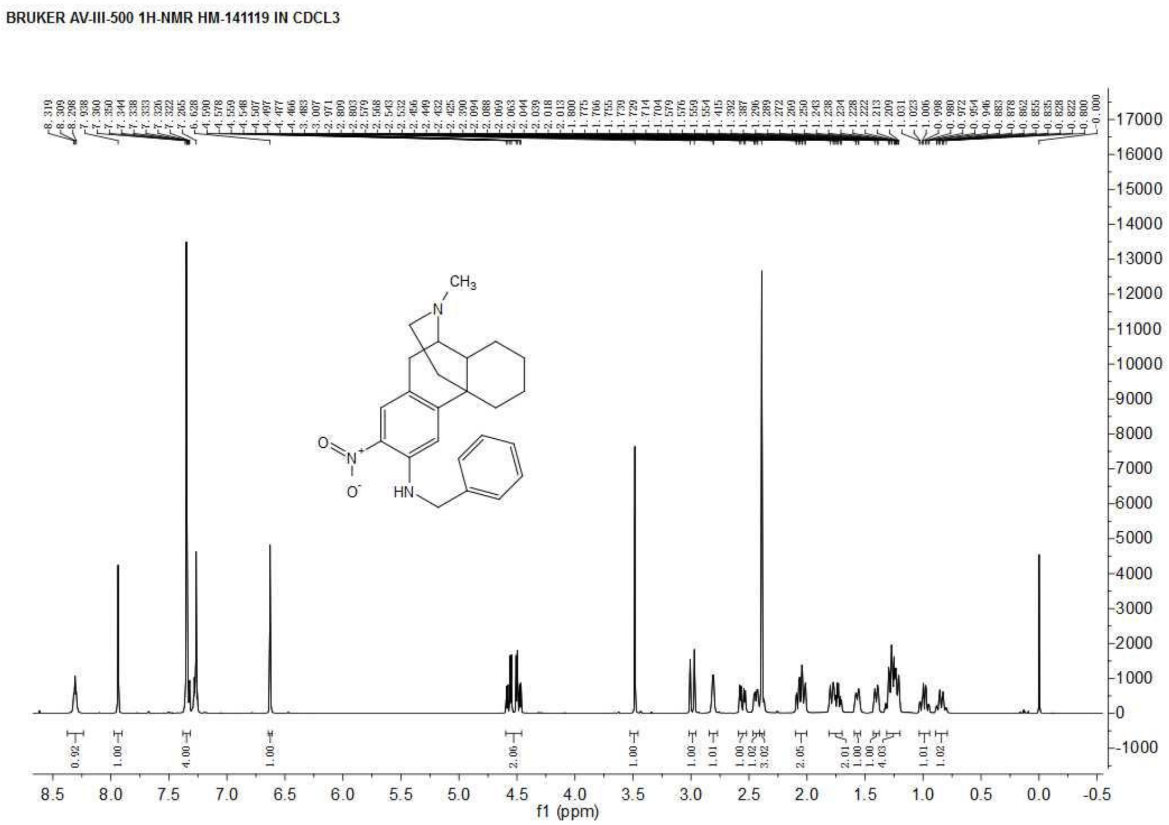

^{1}H NMR spectra of compound 2-nitro-3-benzylamino-N-methylmorphinan (**5**)

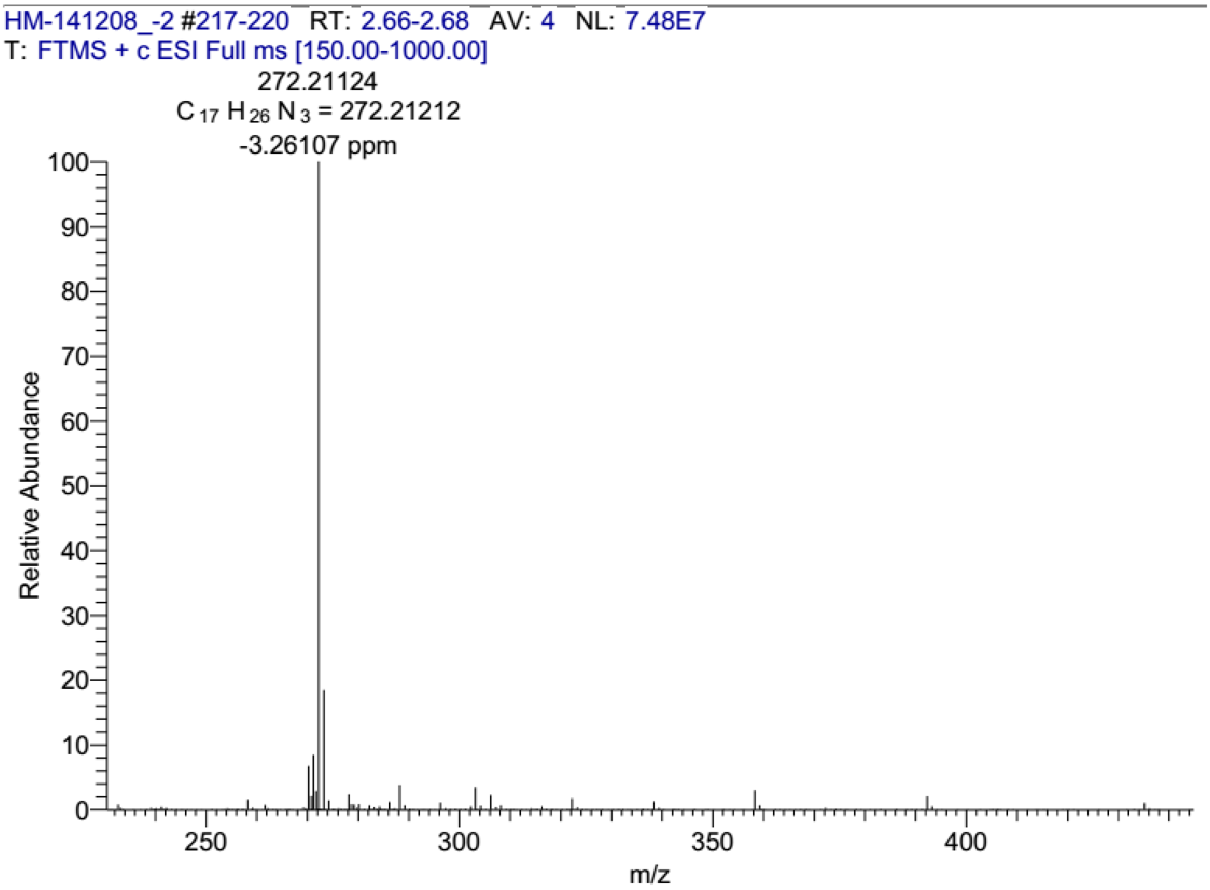

*HRMS spectra of compound N-methylmorphinan-2,3-diamine (**6**)*

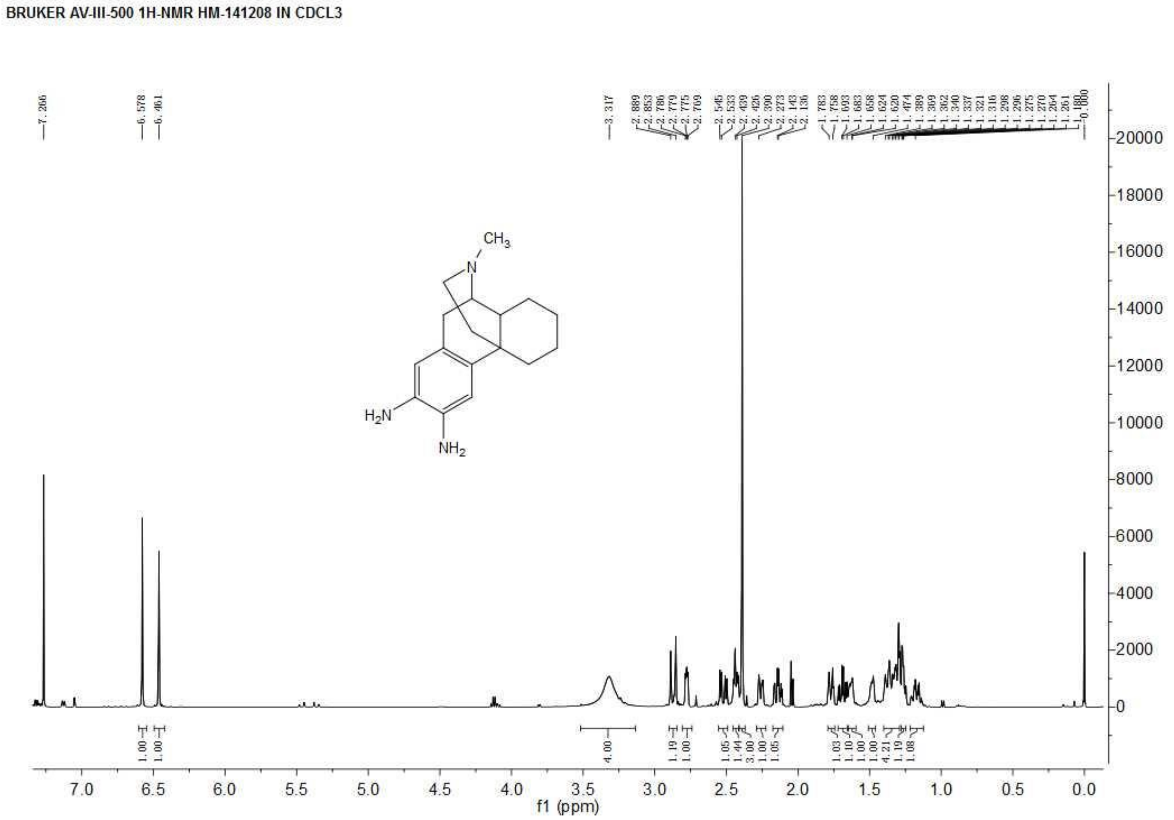

^{1}H NMR spectra of compound N-methylmorphinan-2,3-diamine (**6**)

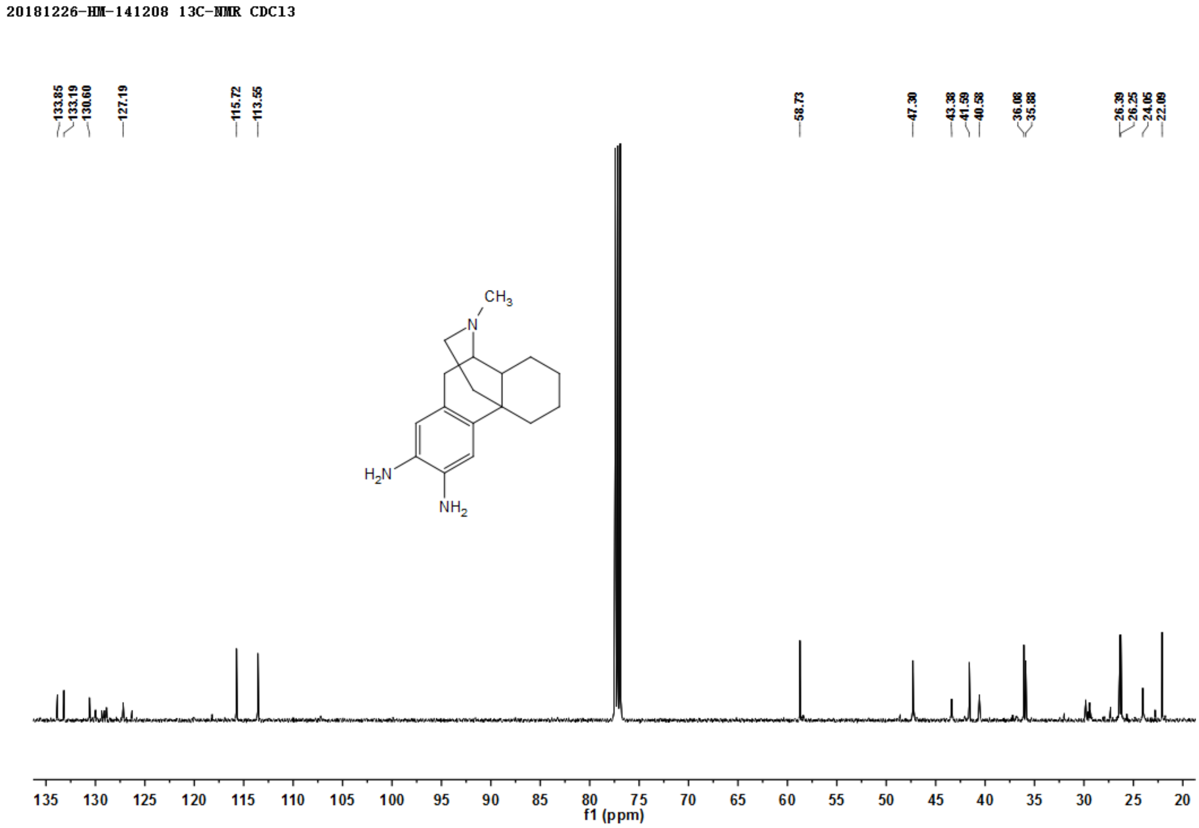

^{13}C NMR spectra of compound N-methylmorphinan-2,3-diamine (**6**)

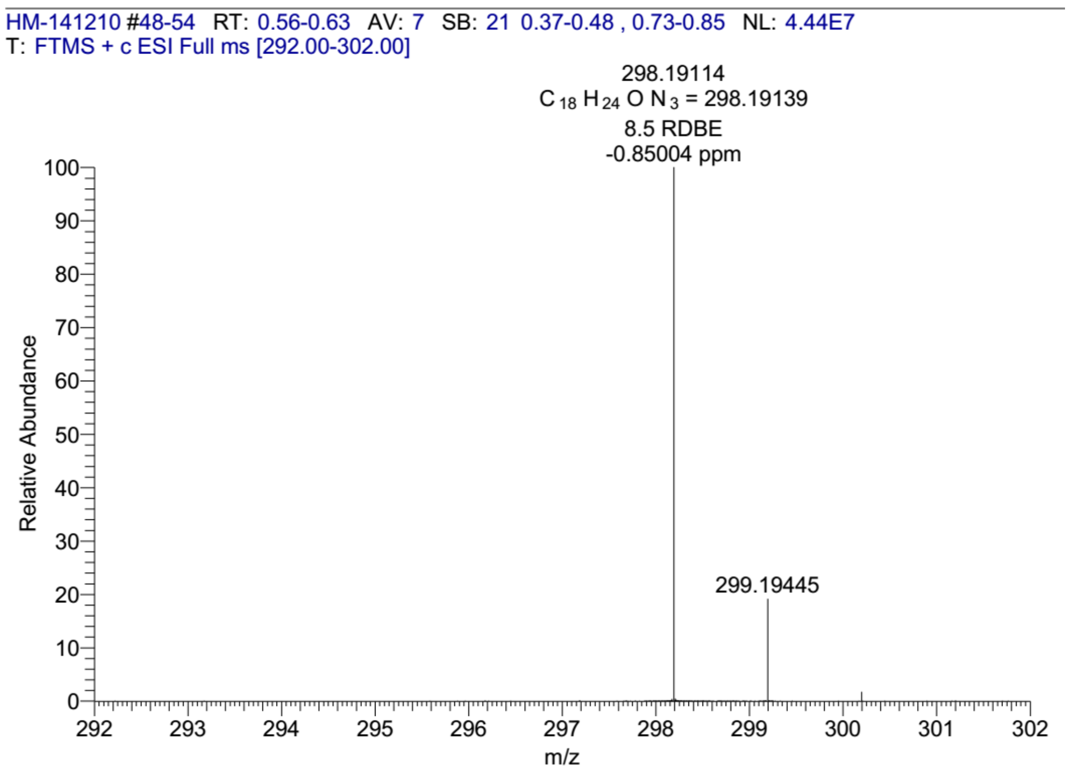

*HRMS spectra of compound imidazolone-N-methylmorphinan (**7**)*

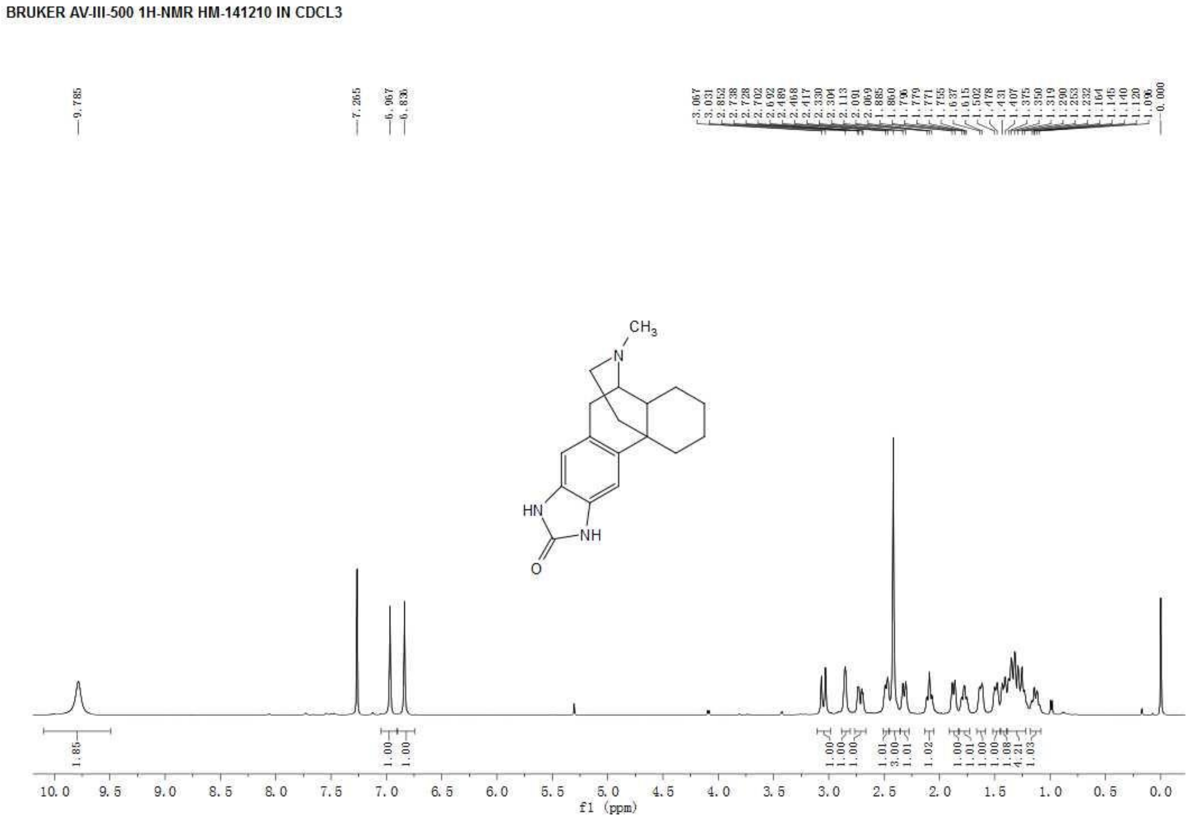

*^{1}H NMR spectra of compound imidazolone-N-methylmorphinan (**7**)*

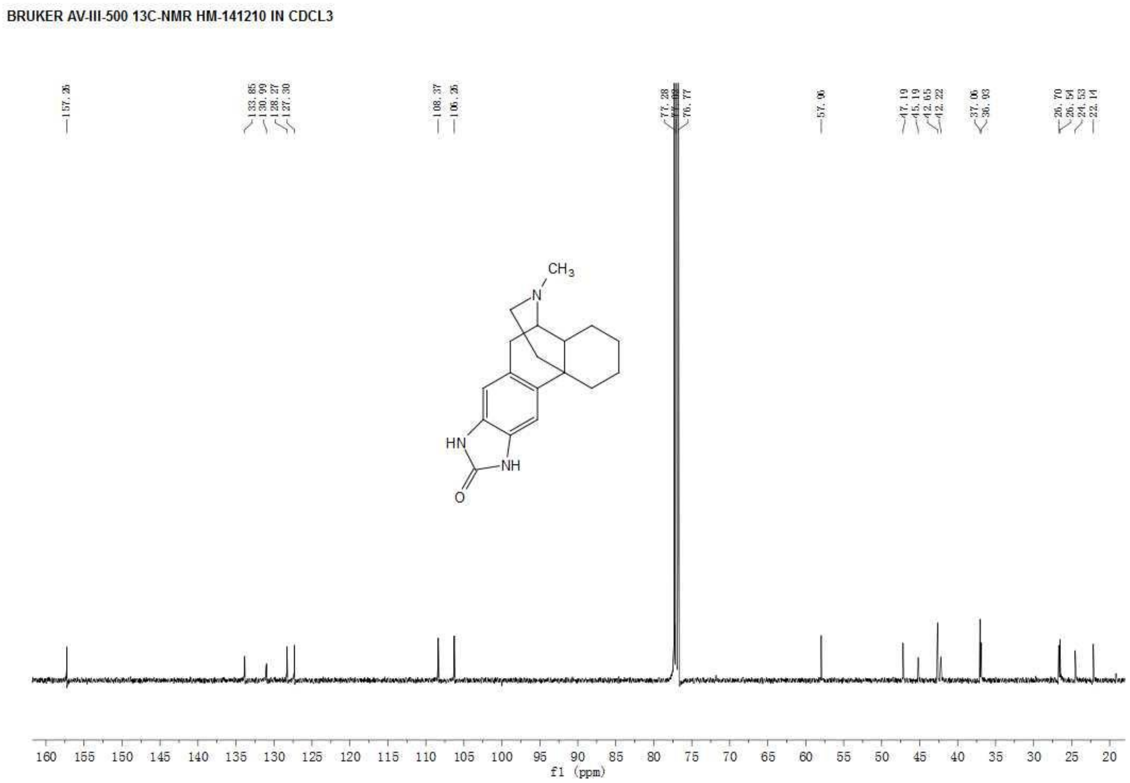

^{13}C NMR spectra of compound imidazolone-N-methylmorphinan (**7**)

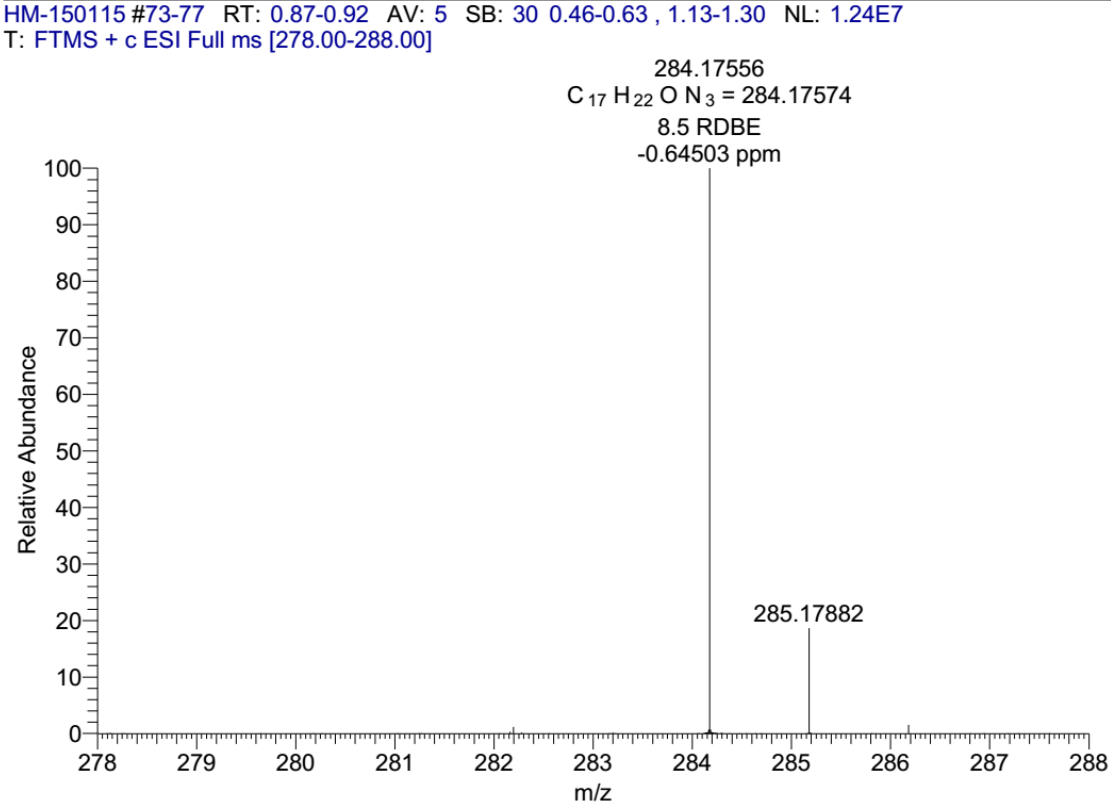

*HRMS spectra of compound imidazolone-morphinan (**8**)*

BRUKER AV-III-500 1H-NMR HM-150309 IN DMSO

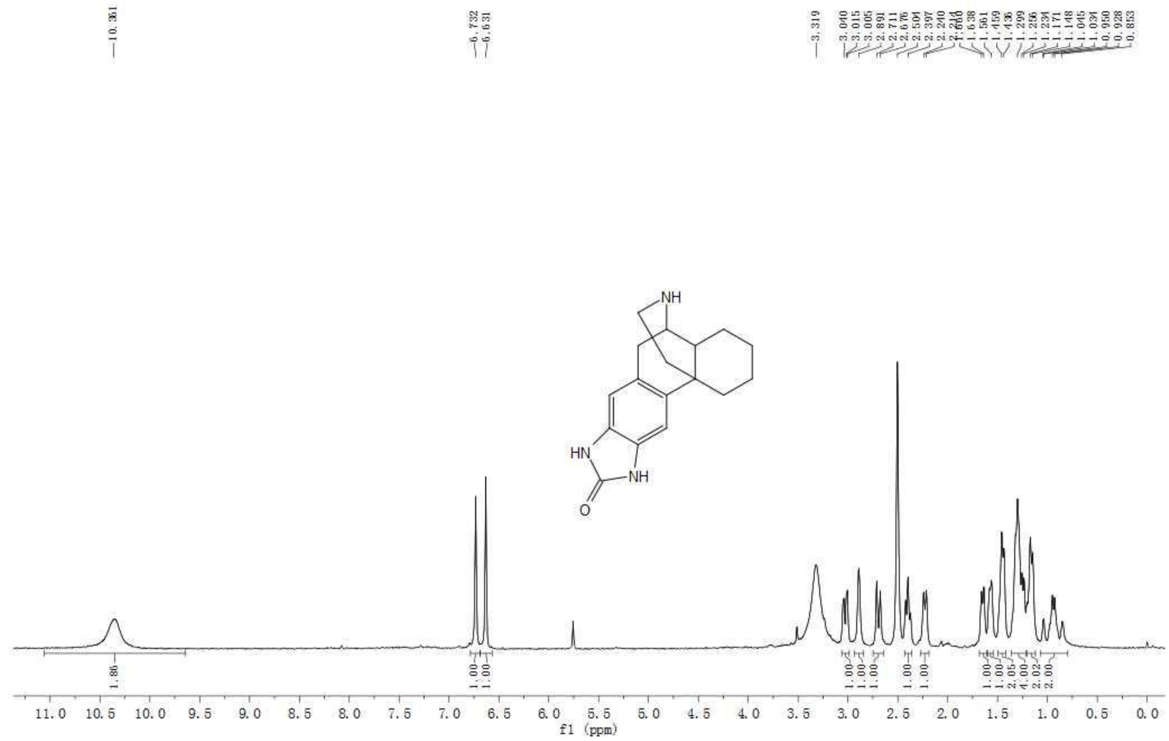

^{1}H NMR spectra of compound imidazolone-morphinan (**8**)

BRUKER AV-III-500 13C-NMR HM-150309 IN DMSO

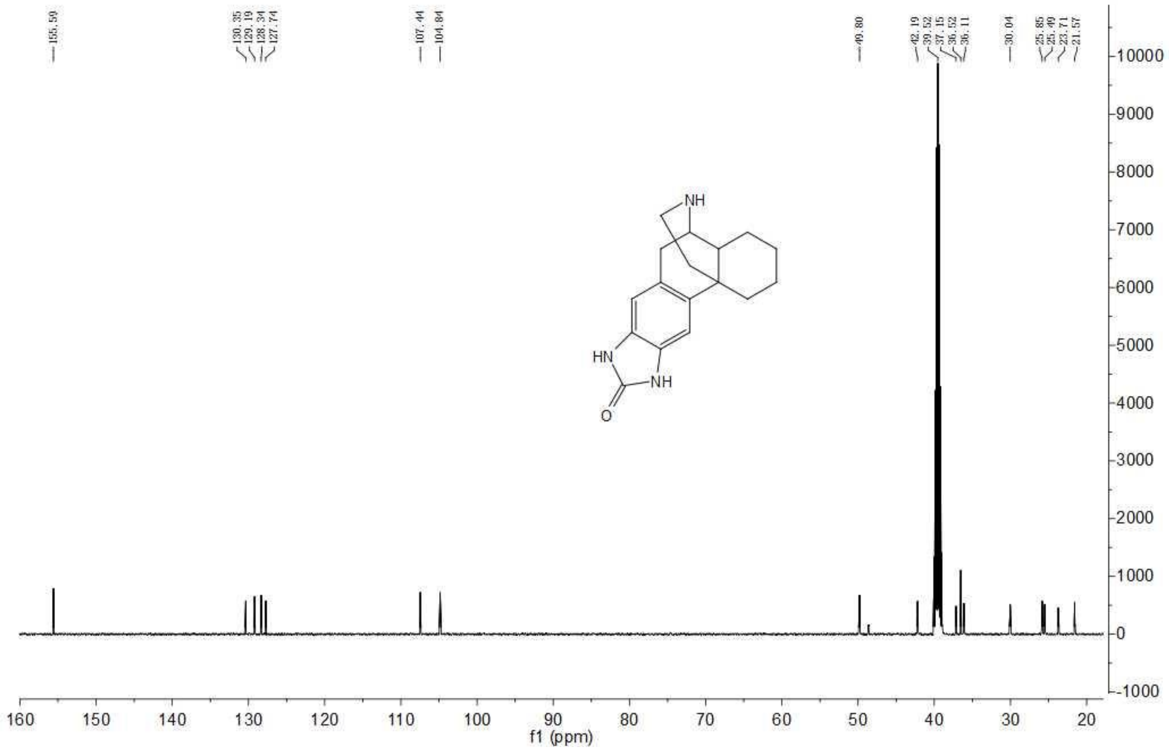

^{13}C NMR spectra of compound imidazolone-morphinan (8)
